# Anther mimicry in an African orchid pollinated by pollen‐feeding beetles

**DOI:** 10.1111/plb.70060

**Published:** 2025-06-23

**Authors:** A. Adit, S. D. Johnson

**Affiliations:** ^1^ Centre for Functional Biodiversity, School of Life Sciences University of KwaZulu‐Natal Pietermaritzburg South Africa; ^2^ Present address: Department of Botany Dr. Harisingh Gour Vishwavidyalaya (A Central University) Sagar Madhya Pradesh India

**Keywords:** Batesian food‐source mimicry, *Disa similis*, floral herbivory, food deception, Orchidaceae, South Africa, visual cues

## Abstract

- Flowers of many species have yellow markings that appear to mimic anthers or pollen and attract the attention of pollen‐seeking insects (usually female bees). We investigated a putative case of anther mimicry in *Disa similis,* an orchid with nectarless mauve flowers and conspicuous yellow markings on the tips of the labellum and lateral petals.
- We studied *D. similis* in grasslands of KwaZulu‐Natal, South Africa, by directly observing floral visitors. Spectral reflectance of floral parts of the orchid and sympatric species were analysed using spectrometry. Pollination success was recorded in relation to colour manipulation and florivory of the petal apices. Overall pollen transfer efficiency and breeding system was estimated to determine pollinator dependence for fruit set.
- The flowers of *D. similis* lack scent and are pollinated by the pollen‐feeding beetle *Isoplia lasiosoma* which chews on the yellow petal apices. Overall flower colour resembles that of co‐flowering plants that are visited by beetles for pollen‐feeding, and the yellow UV‐absorbing colour of the petal apices matches that of anthers and pollen. By covering the yellow markings with purple paint or removing them led to reduced pollination success. Pollen removal and deposition were strongly associated with florivory. Plants are self‐compatible and dependent on pollinator visits for fruit set.
- Previous studies of orchids and other plants that deploy mimicry of anthers and pollen have reported bees or flies as pollinators without physical damage to flowers. Pollination of *D. similis* by pollen‐feeding beetles and their consumption of anther‐like apices of the petals are thus unexpected and novel findings.

## Graphical abstract

Beetles chew the fleshy yellow tips of an orchid's petals and pollinate its flowers, representing a novel case of pollen‐seeking beetles being deceived through anther mimicry.

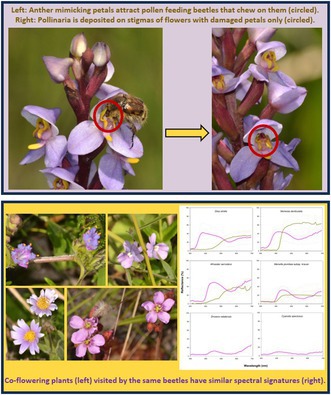

## INTRODUCTION

Plants that lack floral rewards can attract pollinators by imitating their food source, a strategy known as Batesian food‐source mimicry (Schiestl & Johnson 2013). Although both visual and olfactory cues can be involved, visual cues seem to be most important in this type of floral deception (Peter & Johnson 2008; Johnson & Schiestl 2016). This usually involves imitation of the colour, patterning, shape, size and texture of the food source (Roy & Widmer 1999). Colour is crucial for attracting pollinators in most systems of food‐source mimicry, apart from some unusual cases which involve olfaction (Heiduk *et al*. 2023). Studies have shown that floral colour matching between mimics and models significantly influences pollinator preferences and visitation rates (Jersáková *et al*. 2006; Newman *et al*. 2012).

Animals, such as female bees and syrphid flies, that search for pollen rewards are attracted by colour cues (Lunau *et al*. 2024). Plants that are pollinated by pollen‐seeking animals tend to deploy exaggerated visual signals of pollen, regardless of whether they provide actual pollen rewards or are deceptive (Lunau 2002, 2004). This adaptation could be for: (i) defence against pollen collection and consumption, and (ii) higher reward perception and positioning for optimal pollen loading on the pollinator (Lunau 2007). It has been argued that anther/pollen imitation by flowers is the most common form of mimicry in plants, and can be two‐ or even three‐dimensional (Lunau *et al*. 2024). In cases where the anther mimicry involves a three‐dimensional structure, pollinator contact with reproductive structures is often significantly improved (Newman *et al*. 2022). Pollen mimicry is also deployed by some plants with unisexual flowers as a means of attracting pollen‐seeking animals to female flowers (Johnson & Schiestl 2016). In the case of orchids, pollen packaged in pollinaria is not available as a reward, so that orchid flowers must convey the impression of having collectable pollen if they are to rely on pollen‐seeking insects for pollination (Vogel 1978).

Anther/pollen mimicry in Orchidaceae has been recorded as early as the 19^th^ century when Robertson (1887) argued that hairs on the crest of the labellum of *Calopogon parviflorus* resemble dehiscing anthers to attract small bees. Later authors proposed that labellar trichomes/papillae or pseudopollen serve as tactile/visual cues, and may even be collectable despite not offering real nutritional value (Dafni & Ivri 1981; Boyden 1982; Davies *et al*. 2013; Zheng *et al*. 2021). Some orchids also have contrasting nectar guides that may function to give a visual impression of anthers or pollen (Shi *et al*. 2009; Johnson & Schiestl 2016; Ma *et al*. 2016; Lunau *et al*. 2021). Flowers of *Thelymitra* spp. in Australia often have fake anthers derived from column wings or folds that attract pollen‐seeking bees (Bernhardt & Burns‐Balogh 1986; Cropper & Calder 1988).

Beetles account for ~25% of globally identified insect diversity, and are known to play a vital role in plant interaction networks (Ahrens *et al*. 2014; Stork *et al*. 2015). Beetles are the earliest known pollinators of basal angiosperms (Bernhardt 2000), with evidence of flower visitation since the early Cretaceous period (Bao *et al*. 2019). Flowers pollinated by beetles often have fleshy petals to accommodate their propensity for florivory (McCall & Irwin 2006). Orchids with their strongly zygomorphic, side‐facing flowers and pollen packaged in pollinaria have not been considered well‐suited for beetle pollination. Van Der Pijl & Dodson (1966) even suggested that *“there appears to be no trend toward adaptation to beetles as pollinators*” in the orchid family. But in recent years, several studies have included reports of beetle pollination in orchids (Steiner 1998; Peter & Johnson 2006a, 2006b, 2009; Pedersen *et al*. 2013; Sugiura *et al*. 2021), indicating that earlier authors may have been mistaken about the scarcity of beetle pollination in this family.

Flowers of the terrestrial African orchid *Disa similis* Summerh. exhibit a striking contrast between the mostly lavender‐purple/mauve perianth and the bright yellow apices of petals that resemble anthers. The flowers have a pouch‐like spur on the dorsal sepal, which lacks nectar. With up to 15 flowers borne on an erect raceme, they are easily visible in grassland, which also hosts several pollen‐rewarding flowering species. Because its flowers are reminiscent of those of bee‐pollinated *Thelymitra* species, we hypothesized that the flowers of *D. similis* would attract small pollen‐seeking bees that are deceived by the anther‐like visual signal presented by the petals.

The aims of this study were to: (1) identify pollinator(s) of *D. similis* and understand their foraging behaviour, (2) evaluate the functional floral traits responsible for pollinator attraction, (3) assess the correlation between pollinator responses to the yellow petal apices and reproductive success, and (4) document the breeding system of this plant.

## MATERIAL AND METHODS

### Study populations

**Fig. 1:**
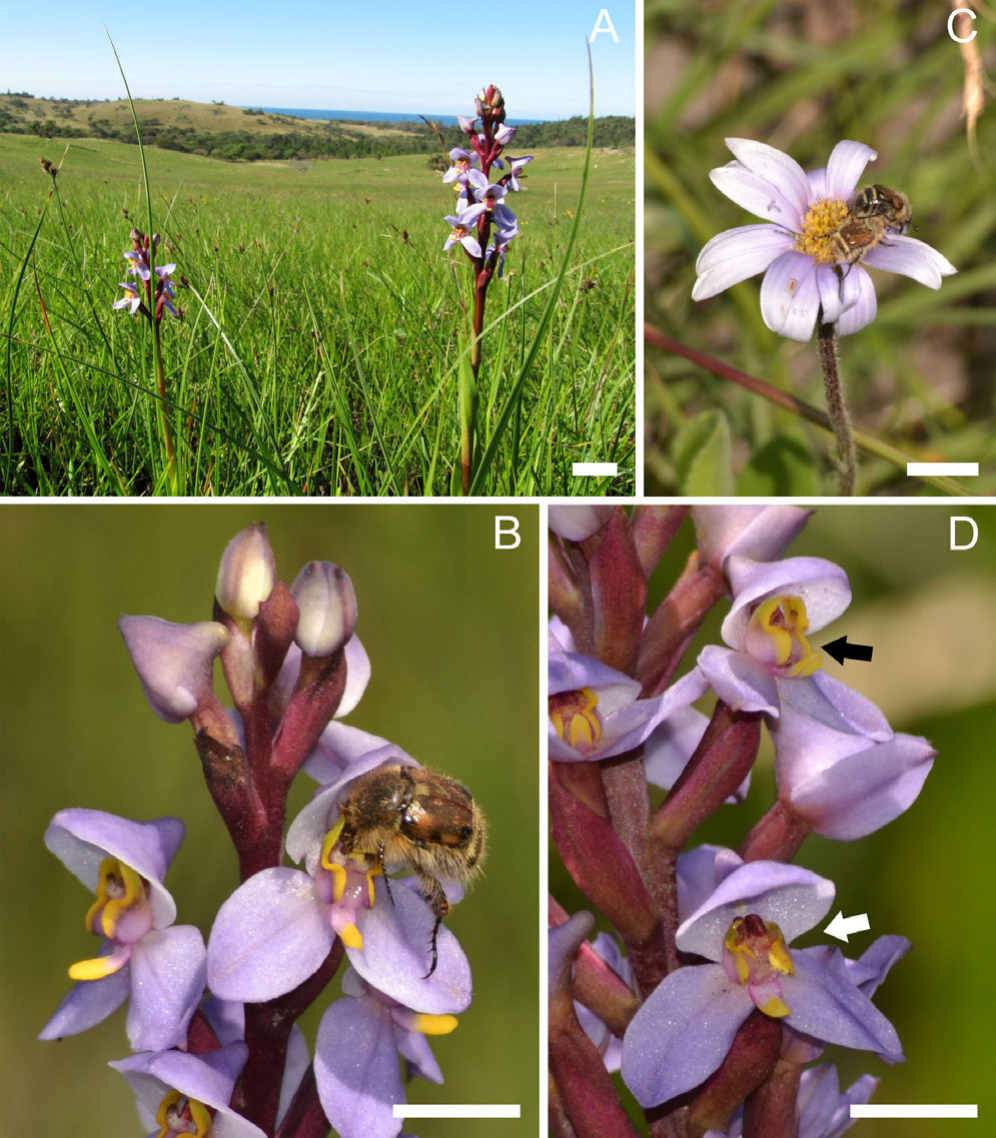
Habitat and flowers of *Disa similis*. (A) Flowering individuals growing in the open grassland of Red Desert Nature Reserve (KZN, SA). (B) *Isoplia lasiosoma* beetle attempting to feed on anther mimicking lateral petals and depositing pollinia on the stigma. (C) The same beetle species visiting flowers of *Afroaster serrulatus* co‐flowering with *D. similis*. (D) While no evidence of pollinia removal or deposition was found on undamaged flowers (black arrow) of *D. similis*, flowers with damaged lateral petals have pollen massulae deposited on the stigma (white arrow). Scale: 1 cm.

*Disa similis* (Orchidoideae: Diseae: Disinae) is a terrestrial orchid native to South Africa, Angola and Zambia. The plants emerge in August and bloom between September and November, with peak flowering in October. In South Africa, the species is known to occur in swamps and marshy grasslands along the coastal region of KwaZulu‐Natal and the Eastern Cape (Fig. 1A). Our study was conducted using three populations: (i) Umtamvuna Nature Reserve (UNR; 30.993693° S, 30.159070° E; total 16 individuals observed in 2023 and 11 in 2024), (ii) Red Desert Nature Reserve (RDNR; 31.067552° S, 30.195898° E; total 29 individuals observed in 2023 and 24 in 2024) and (iii) Rennie's Beach (RB; 31.070955° S, 30.206204° E; total 20 individuals observed in 2023 and 17 in 2024). A representative herbarium specimen for *D. similis* (NU0095947) was deposited in the Bews Herbarium (NU) at the University of KwaZulu‐Natal.

### Insect visitors

To identify pollinators, we conducted observations in September and October 2023 and 2024 for a total of 170 h over 28 days. We made direct observations between 09:00 h and 17:00 h. Some of the flower visitors carrying pollinaria (*n* = 10) were captured and subsequently pinned for record and identification. To confirm the pollen‐feeding habit of the pollinator, mouth parts of captured insects were swabbed with fuchsin gel (Beattie 1971) and mounted on glass slides to check for pollen grains of co‐flowering plant species. This was done by comparing our samples with reference slides from pollen of co‐flowering plant species. This was further tested by analysing droppings of the captured beetles (*n* = 8) for pollen, by crushing them in a drop of water on glass slides and viewing under a light microscope.

### Natural pollination success

The number of massulae per pollinium (*n* = 10) was counted under a stereo microscope. In addition, we recorded the number of pollinaria removed, massulae deposited and the incidence of chewed petals for 110 flowers from 10 individuals (sampled in all three populations in both flowering seasons). For this, three individuals were used from UNR, four from RDNR and three from RB. Swollen hard ovaries of older wilting flowers were considered an indication of fruit set. Data from these 110 flowers was pooled to calculate pollen transfer efficiency as PTE = D/MR, where D is total number of massulae deposited on stigma, M is average number of massulae per pollinium, and R is total number of pollinaria removed by the pollinator (Johnson *et al*. 2005).

### Effects of yellow markings and florivory on pollination

To check if the yellow colour of petals plays a role in attracting potential pollinators, we conducted experiments where (i) these portions were painted with a UV absorbing colour matching the sepals (following consumables and instrumentation in Jersáková *et al*. 2012; Fig. S1), and (ii) these portions were excised using a blade. This was done on 12 individuals (*n* = 72 flowers); with six flowers treated on each individual, two with petals painted, two with petals excised and two unmanipulated (control) (data pooled from all three populations). These flowers were then tagged and checked 2 weeks later if they set fruit. Comparisons of the frequencies of fruit set among treatment groups were made with Fisher's exact test implemented in Omnicalculator (https://www.omnicalculator.com/statistics/fishers‐exact‐test).

To assess whether pollination success in the sample of 110 flowers on 10 plants that were surveyed for pollination success was correlated with flower damage (when little or no yellow was visible) we used Generalized Estimating Equations (GEEs) implemented in SPSS v. 29 (IBM). We used a negative binomial distribution with a log link function for counts of massulae deposited on the stigma, and a binomial distribution and logit link function for the proportions of pollinaria removed and flowers that set fruit. Plant individual was treated as a grouping factor (with an exchangeable correlation matrix) to account for potentially statistically correlated responses among flowers on each plant. Flower damage was treated as a fixed effect and flower position from the bottom of each inflorescence was included as a covariate in the model to control for potential effects of flower age (and thus time of exposure) when considering the relationship between flower damage and pollination success.

### Floral traits

To quantify floral longevity and timing of anthesis, we tagged flowers (*n* = 10 flowers; 10 individuals) at bud stage, checked for anthesis and tracked them until they senesced through direct observation.

Our preliminary observations suggested that the contrasting colour of the petal apices might reflect mimicry of anthers or pollen or both. Therefore, spectral reflectance of the sepals and petal apices of *D. similis*, and the petals and anthers of co‐occurring plant species was measured using an Ocean Optics USB 2000 spectrometer (Dunedin, FL, USA) and Ocean Optics DT‐mini deuterium tungsten halogen light source (200–1100 nm) as described by Johnson & Andersson (2002). We determined the spectral reflectance over the UV–visible range (300–700 nm) using five flowers (from five different individuals for each species; total five species from all three populations).

Flowers of *D. similis* are odourless to humans. To confirm this, we conducted headspace extraction using a PAS500 Personal Air Sampler (Spectrex, CA, USA) to extract volatiles emitted by inflorescences of three plants (each with 15 open flowers) enclosed in Nalophan polyacetate bags (Kalle, Germany) for 2 h at noon. Volatiles were collected in glass cartridges containing 1 mg each of carbotrap B (20–40 mesh; Sigma‐Aldrich) activated charcoal and tenax TA (60/80; Supelco). We also took samples from empty bags (control; two replicates) to ascertain and omit environmental contaminants. Subsequently, all samples were stored at −20 °C until Gas Chromatography–Mass Spectrometry (GC–MS) analysis using a Varian CP‐3800 gas chromatograph coupled to a Varian 1200 quadrupole mass spectrometer fitted with a Varian 1079 PTV injector port modified with a ChromatoProbe thermal desorption device into which sample cartridges were placed. The GC temperature programme conditions used followed Johnson *et al*. (2020a). Compounds were checked using Varian MS Workstation (v. 7.0) and NIST MS Search (v. 2.3) with the NIST 2017 mass spectral library.

### Breeding system

Pollinator exclusion experiments were performed on randomly selected bagged virgin flowers in four treatments (58 flowers in total): (i) emasculated flowers (*n* = 12) to test for apomixis; (ii) unmanipulated flowers (*n* = 12) to test for spontaneous selfing; (iii) manually self‐pollinated flowers (*n* = 18) to test for self‐compatibility; (iv) cross‐pollinated flowers (*n* = 18) as a positive control. Furthermore, we also tagged and monitored unmanipulated flowers (*n* = 30) that were not bagged in order to estimate fruit set arising from open pollination. Six plant individuals were used to conduct exclusion experiments, with each individual subjected to all treatments in a balanced design. Seeds per fruit resulting from each treatment were estimated before capsules dehisced (1 month after performing the experiments) to ascertain the efficacy of pollination treatments (*n* = 10 fruits). Fruit set and seed‐set data were compared using generalized linear models (GLMs) incorporating a binomial distribution and logit link function in SPSS v. 29 (IBM).

## RESULTS

### Insect visitors

Flowers of *Disa similis* were visited and pollinated only by individuals of the beetle *Isoplia lasiosoma* (Scarabaeidae: Rutelinae: Anomalini: Isopliina), which were observed to chew on the tips of the lateral petals (Fig. 1B). A total of 10 of these beetles were observed on the flowers and, because this beetle species has been rarely observed, and collected only once previously (leading to their original description), the specimens were submitted to the South African National Collection of Insects (SANC, Pretoria; COLS17224–COLS17226). These beetles spent long periods (median = 22 min) on each flower (*n* = 11), slowly chewing on the yellow petal apices. The beetles aligned perfectly with the orchid's reproductive structures, ensuring pollinaria withdrawal and massulae deposition on the underside of the thorax (observed five times). Beetles moved among flowers on the same inflorescence and were also seen to move to different individuals. The beetles visited flowers between 10:30 h and 16:30 h. The beetles were also observed feeding on anthers/pollen of certain co‐flowering species in the study site *viz*., *Afroaster serrulatus* (Asteraceae), *Cyanotis speciosus* (Commelinaceae), *Drosera natalensis* (Droseraceae) and *Merwilla plumbea* (Asparagaceae), which were also visited frequently by several native bees (Fig. 1C). The beetles were also seen on the flowers of *Nemesia denticulata* (Scrophulariaceae); however, no evidence of pollen‐feeding was observed on that species. These beetle visits to co‐flowering plants also lasted up to 20 min per flower (*n* = 15). Microscopy investigation of swabs taken from mouthparts of beetles collected on *D. similis* (*n* = 10) showed pollen grains of these co‐flowering species. Slide preparations of beetle droppings also revealed pollen grains of co‐flowering species, confirming the pollen‐feeding behaviour of the beetle (Fig. S2).

### Natural pollination success

Only 14.5% of flowers had their pollinia removed, while 16.4% flowers had massulae deposited on the stigma, all of which formed fruits (*n* = 110 flowers; 10 individuals). Each pollinium had an average of 420 ± 1.97 massulae (*n* = 10 flowers). Pollen transfer efficiency was calculated as 0.731.

### Effects of yellow markings and florivory on pollination

Although flowers which had their petal apices painted (*n* = 24) or excised (*n* = 24) did not set any fruits, the unmanipulated controls produced seven fruits (*n* = 24). This difference in fruit set between the unmanipulated controls and the former two treatment groups pooled was statistically significant according to a two‐tailed Fisher's exact test (*P* = 0.0002).

Flowers that showed signs of chewing of the petals and labellum apices also showed evidence of pollen removal and pollen deposition (*n* = 110 flowers, 10 individuals; Fig. 1D). Pollination success was not observed in flowers that showed no signs of petals being chewed (Table 1).

**Table 1:** Measures of pollination success in intact versus chewed flowers of *Disa similis* studied in KwaZulu‐Natal, South Africa.

measure	petals intact	petal apices damaged	*χ* ^2^	*P*
Pollinaria removed	0.00 ± 0.00	0.38 ± 0.073	7.41	0.006
Massulae deposited	0.00 ± 0.00	233.05 ± 21.89	7.78	0.005
Fruit set	0.00 ± 0.00	0.62 ± 0.056	7.67	0.006

Values are mean (± SE) and represent the proportion of pollinaria removed, counts of massulae deposited on stigmas and the proportion of flowers that set fruit. Sample size was 110 flowers on 10 plants.

Analyses showed that after accounting for flower position statistically, there were very strong positive associations between damage to lateral petals and pollinia removal (*χ* ^2^ = 7.41, *P* = 0.006), massulae deposited (*χ* ^2^ = 7.78, *P* = 0.005) and fruit set (*χ* ^2^ = 7.67, *P* = 0.006). In all these analyses, flower position did not have significant effects on measures of pollination success.

### Floral traits

**Fig. 2:**
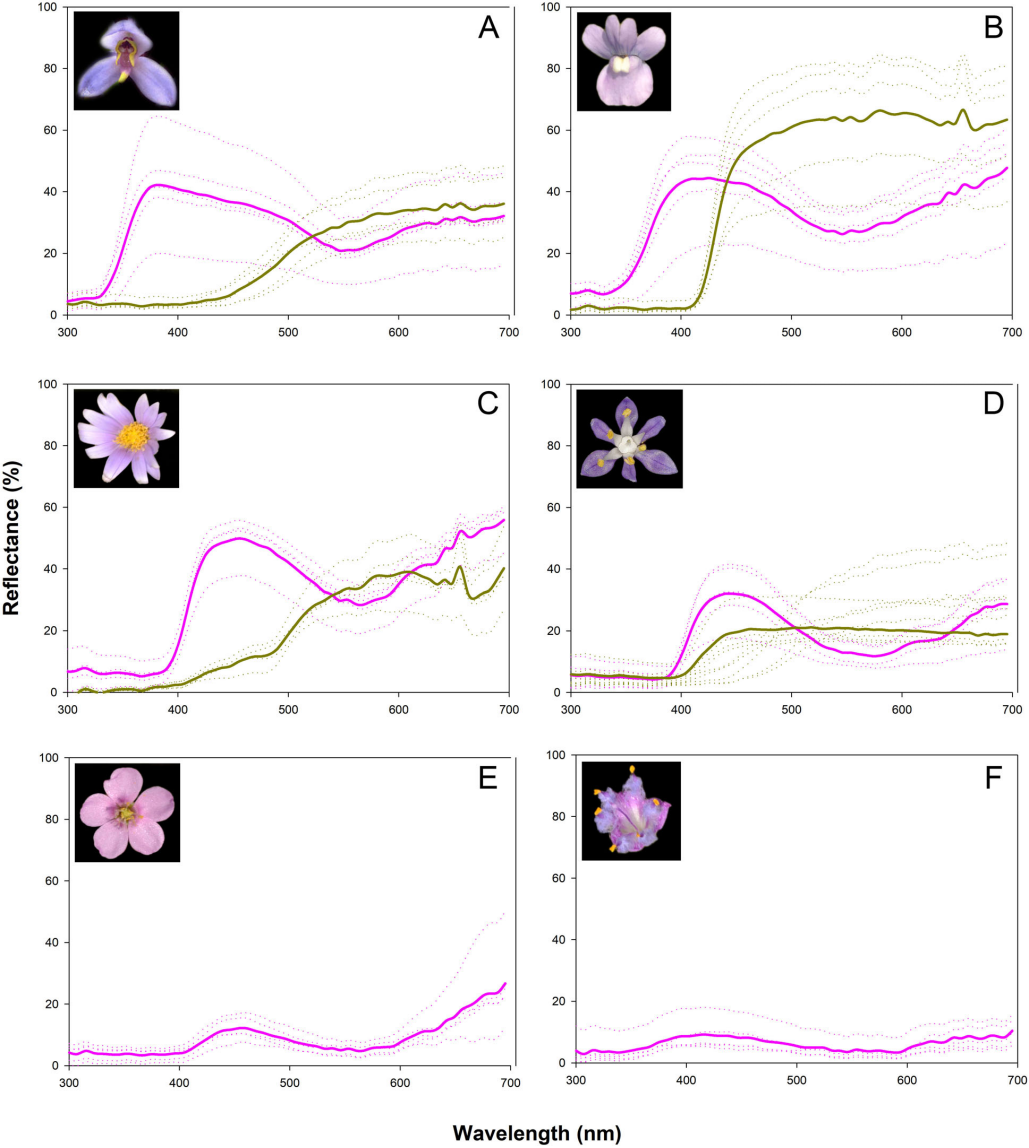
Spectral reflectance of different floral parts in (A) *Disa similis*, (B) *Nemesia denticulata*, (C) *Afroaster serrulatus*, (D) *Merwilla plumbea*, (E) *Drosera natalensis* and (F) *Cyanotis speciosus*. Purple lines denote reflectance of sepals in the case of A, and petals for B–F. Dark yellow lines denote reflectance of petals in the case of A, “nectar guide” for B, and anthers/pollen for C and D. Reflectance of anthers/pollen for E and F could not be measured. The dashed lines represent reflectance spectra of individuals and the solid lines depict averages of these spectra.

Flowers open during the morning and last for up to 15 days if not pollinated (*n* = 10 flowers; 10 individuals). Spectral data suggest that sepals and petals of *D. similis* have similar reflectance to petals and anthers of co‐flowering plants (Fig. 2). These co‐flowering plants were also observed to be visited by *Isoplia lasiosoma* beetles. Analysis of headspace samples revealed no volatile compounds in control samples, indicating that flowers of *D. similis* are effectively unscented.

### Breeding system

No fruits were set in emasculated and intact bagged flowers, indicating that the species is pollinator‐dependent. Fruit set did not differ between manually self‐ and cross‐pollinated flowers (Table 2). Fruit set in naturally pollinated flowers was about five‐fold lower than that in hand‐pollinated flowers, but no significant difference was observed in seed set among these treatments (Table 2).

**Table 2:** Results of controlled pollination experiments conducted on *Disa similis*.

measure of fecundity	emasculated	unmanipulated	hand self‐pollinated	hand cross‐pollinated	open pollinated	χ^2^	*P*
Fruit set (%)	0.0 ± 0.0^a^ (12)	0.0 ± 0.0^a^ (12)	65 ± 13.1^b^ (18)	78 ± 10.7^b^ (18)	11 ± 5.9^c^ (30)	8.662	<0.001
Seed set (%)	‐	‐	76.8 ± 0.4^a^ (10)	77.2 ± 0.4^a^ (10)	77.3 ± 0.5^a^ (10)	0.612	0.549

Values sharing the same letter superscripts are not significantly different. Values within parentheses denote flowers manipulated for each treatment. Six individuals were used to conduct exclusion experiments; each individual was subjected to all treatments.

## DISCUSSION

The present study shows that *D. similis* is pollinated by pollen‐feeding beetles that are seemingly attracted to (and chew on) the yellow petal apices. The yellow apices of the petals can be interpreted as an example of anther mimicry, as they visually resemble anthers of nearby plants which are foraged for pollen by *Isoplia lasiosoma* beetles, and because the petal apices are chewed by these beetles, which seem to be specialized pollen‐feeders. We showed that there is a strong association between petal damage by chewing and pollinia removal and deposition of pollen massulae. While the orchid is self‐compatible, it relies on pollinator visits for fruit set. To our knowledge, this is the first report showing involvement of beetles in the pollination of a plant that employs anther mimicry.

Beetles are well known as pollinators in South Africa and have been reported to pollinate species in a few orchid genera *viz*. *Ceratandra* (Steiner 1998), *Eulophia* (Peter & Johnson 2006b, 2014), *Orthochilus* (Peter & Johnson 2006a, 2009) and *Satyrium* (Johnson *et al*. 2007, 2011). Within the *Disa* clade, two other species have been reported to be beetle‐pollinated: the sexually deceptive: *D. forficaria* pollinated by longhorn beetles (Cohen *et al*. 2021) and the nectar rewarding *D. elegans* pollinated by fruit‐chafer beetles (Liltved & Johnson 2012). Monkey beetles (Hopliini) have also been observed to carry pollinaria of *D. tenuifolia*, but this species is pollinated primarily by megachilid bees (Johnson & Steiner 1994). Unlike most examples of beetle pollination in orchids, where floral scent acts as a key incentive for pollinator attraction (Wallace 1980; Singer & Cocucci 1997; Johnson *et al*. 2007; Peter & Johnson 2014; Cohen *et al*. 2021), *D. similis* appears to rely solely on visual cues, namely petals that mimic the shape and colour of anthers of co‐flowering species. Previous studies have also established that some scarab beetles can differentiate flowers based on visual cues alone (Dafni 1997; Johnson & Midgley 2001; Van Kleunen *et al*. 2007).

It is thought that beetles prefer to feed on pollen and perianth tissue (Scaccabarozzi *et al*. 2020; Lunau *et al*. 2021), rather than nectar; and that this is a primitive trait (Gutowski 1990), as their mouthparts have not evolved to access rewards that are usually concealed in flowering plants (but see Johnson *et al*. 2007). Goldblatt & Manning (2011) suggest the embedding behaviour of beetles results in a higher degree of floral damage, and that this often occurs in generalist flowers. This aligns with our study, as *D. similis* resembles co‐flowering plants (models) that have open flower structures. Low natural fruit set indicates that beetle visits are relatively rare. However, comparable seed‐set between naturally‐pollinated and hand‐pollinated flowers suggests that the beetles deposit sufficient numbers of massulae on the stigma. High PTE (73.1%) in the present study probably reflects predominant geitonogamy. Extraordinarily high PTE values are not uncommon in some orchids (Harder & Johnson 2008; Johnson & Harder 2023), as evidenced in case of the beetle‐pollinated species *Orthochilus ruwenzoriensis*, which has been reported to have PTE of 68% (Singer & Cocucci 1997). There is also strong evidence of pollinator limitation from the fruit set results comparing open‐pollination and cross‐pollination. Although these beetles clearly pollinate the flowers, they are not common, and may disperse mostly self‐pollen. Flowers of *D. similis* seem to have adapted to vector‐dependent self‐pollination since there is no evidence of mechanisms to prevent self‐pollination through geitonogamy, such as prolonged pollinium bending, in this orchid species.

Beetles in the subfamily Rutelinae have been labelled as phytophagous (general herbivores), commonly observed feeding on partially decomposed wood and accumulated detritus (García‐Atencia *et al*. 2024). Some beetles of the Anomalini tribe are even reported to be root‐eating (Pardo‐locarno *et al*. 2012). *Isoplia* spp. resemble monkey beetles (Hopliini) in their overall appearance (Ahrens *et al*. 2014) and have an unresolved taxonomic position (oscillating between Melolonthinae and Rutelinae). Besides their general appearance, they also deviate from other Ruteline beetles due to their “*adaptation to floral life*” (Peringuey 1902), a behavioural trait that is more in line with hopline beetles. It is unclear whether the *I. lasiosoma* beetles found to pollinate *D. similis* obtain nutritional benefit from chewing on the petal apices. This beetle is the only species in the genus that is known from South Africa. The two other species in the genus are found further north in Africa (Machatschke 1960), and there is insufficient information on the ecology and feeding preferences of this group to determine if they are florivores, pollenivores or general herbivores. Our own observations indicate that the beetles are specialist pollen feeders and, in addition to the list of host flowers reported here, they have also been observed feeding on pollen of *Leucadendron spissifolium* (Proteaceae) at Umtamvuna (Renzo Perissinotto pers. com.).

Beetles are known to feed on petals as general herbivores, besides also being pollenivores of specific plant species (Goldblatt & Manning 2011). It is thus unclear whether the pollination of *D. similis* should be considered deceptive. To the extent that the beetles misclassify the yellow petal apices as anthers and chew into them in search of pollen, the system can be considered deceptive, but we cannot exclude the possibility that the behaviour is general florivory, although in the latter case we would also expect damage to other parts of the perianth and this was not the case, nor did we see evidence of petal damage from these beetles on other flowers in the same community. There are other cases of apparent floral mimicry where pollinators obtain some kind of reward from flowers, and yet authors have concluded that they are effectively deceptive due to cognitive misclassification by the visitors. Such examples include some fly‐pollinated plants such as *Ceropegia gerrardii* (protein rich droplets; Heiduk *et al*. 2023), *Orbea lutea* (nectar; Shuttleworth *et al*. 2017), *Wurmbea* spp. (nectar; Johnson *et al*. 2020b) and *Gorteria diffusa* (nectar and pollen; Ellis & Johnson 2010).

Even though beetle damage to petals in the present case represents florivory, it is most likely a case of cognitive misclassification (mistaking the yellow pollen apices for pollen) as the damage was limited to the yellow petal apices and not the rest of the perianth, which is presumably identical in terms of nutritive content. Nevertheless, florivory is known to have effects on plant fitness ranging from positive (McCall & Irwin 2006), through neutral (Malo *et al*. 2001), to directly and indirectly negative (Mothershead & Marquis 2000; Haas & Lortie 2020; Adit *et al*. 2022). Our study is one of the few cases where floral herbivory enhances pollination (Hillier *et al*. 2018; Tan & Tan 2018; Cardoso *et al*. 2021).

In contrast to the previous studies where anther/pollen mimics are pollinated by bees, we present a novel case where the lateral petals and labellum of *D. similis* mimic anthers and attract pollen‐feeding beetles. As the flowers are unscented, plants rely on visual cues to deceive the pollinator. We also show that damage to anther‐mimicking petals by beetles is associated with pollen removal/deposition and fruit set. It is likely that pollen‐feeding beetles play a role in pollination of *D. similis* populations outside of South Africa, as *D. similis* flowers in Angola have been photographed with partially missing (chewed) lateral petals (https://www.inaturalist.org/taxa/584033‐Disa‐similis). Direct observations in these populations, along with more studies on feeding habit of *I. lasiosoma*, would more firmly demonstrate anther mimicry and floral deception in *D. similis*.

Despite beetles being one of the most diverse group of animals on Earth, only a small proportion have been reported to carry out pollination. For some time, it was believed that plants were poorly adapted for pollination by coleopterans as compared to hymenopterans and lepidopterans (Kevan & Baker 1983); however, this paradigm has been countered by several studies (Bernhardt 2000; Cohen *et al*. 2021). It would be interesting to explore whether the similarity between the general coloration of petals of *D. similis* and those of sympatric plants (Fig. 1) is part of a more general mimicry strategy involving imitation of the signals of plants used by the beetles as food sources. As beetles are known to consume pollen, we expect that many more examples of plants that employ anther/pollen mimicking signals to attract pollen‐feeding beetles for pollination will be discovered.

## AUTHOR CONTRIBUTIONS

AA: Conceptualization, data collection, data curation, data analysis, writing – original draft and editing. SDJ: Conceptualization, data analysis, writing – editing.

## CONFLICT OF INTEREST

The authors declare no competing interests.

## Supporting information


**Fig. S1.** **S**pectral reflectance of purple paint used to match spectral reflectance of purple paint used to match spectral reflectance of *Disa similis* sepal (see Fig. 2A for comparison) to check response of beetles when there are no yellow markings on the flower.
**Fig. S2.** Droppings from six captured *Isoplia lasiosoma* beetles clearly reveal visible pollen grains (red arrows) of plant species co‐flowering with *Disa similis* in the Red Desert Nature Reserve and Rennie's Beach (KZN, SA) under a compound microscope. Scale: 0.1 mm.

## Data Availability

All data generated during the study are included in the manuscript and the supplementary file.
