# Supplementary material for: Anther mimicry in an African orchid pollinated by pollen‐feeding beetles
Source: Plant Biol (Stuttg). 2025 Jun 23;27(6):1071–8. doi: 10.1111/plb.70060 (PMC12477311; doi:10.1111/plb.70060)
Supplement: Supplementary file 1 — Fig. S1. Spectral reflectance of purple paint used to match spectral reflectance of purple paint used to match spectral reflectance of Disa similis sepal (see Fig. 2A for comparison) to check response of beetles when there are no yellow markings on the flower. Fig. S2. Droppings from six captured Isoplia lasiosoma beetles clearly reveal visible pollen grains (red arrows) of plant species co‐flowering with Disa similis in the Red Desert Nature Reserve and Rennie's Beach (KZN, SA) under a compound microscope. Scale: 0.1 mm. [file PLB-27-1071-s001.pdf]

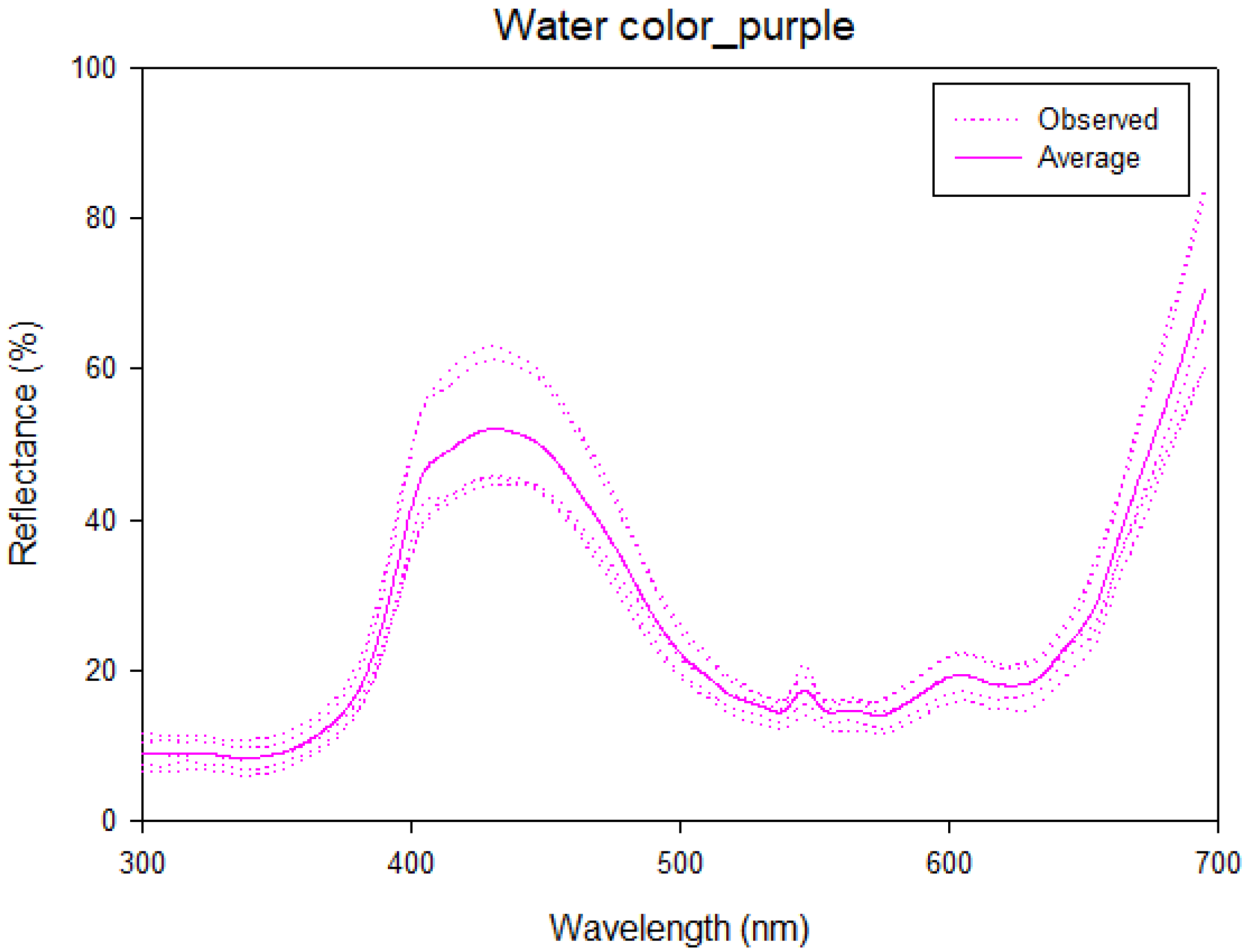

Supplementary fig. 1. Spectral reflectance of purple paint used to match spectral reflectance of *Disa. similis* sepal (see Fig. 2A for comparison) to check response of beetles when there is no yellow markings on the flower.

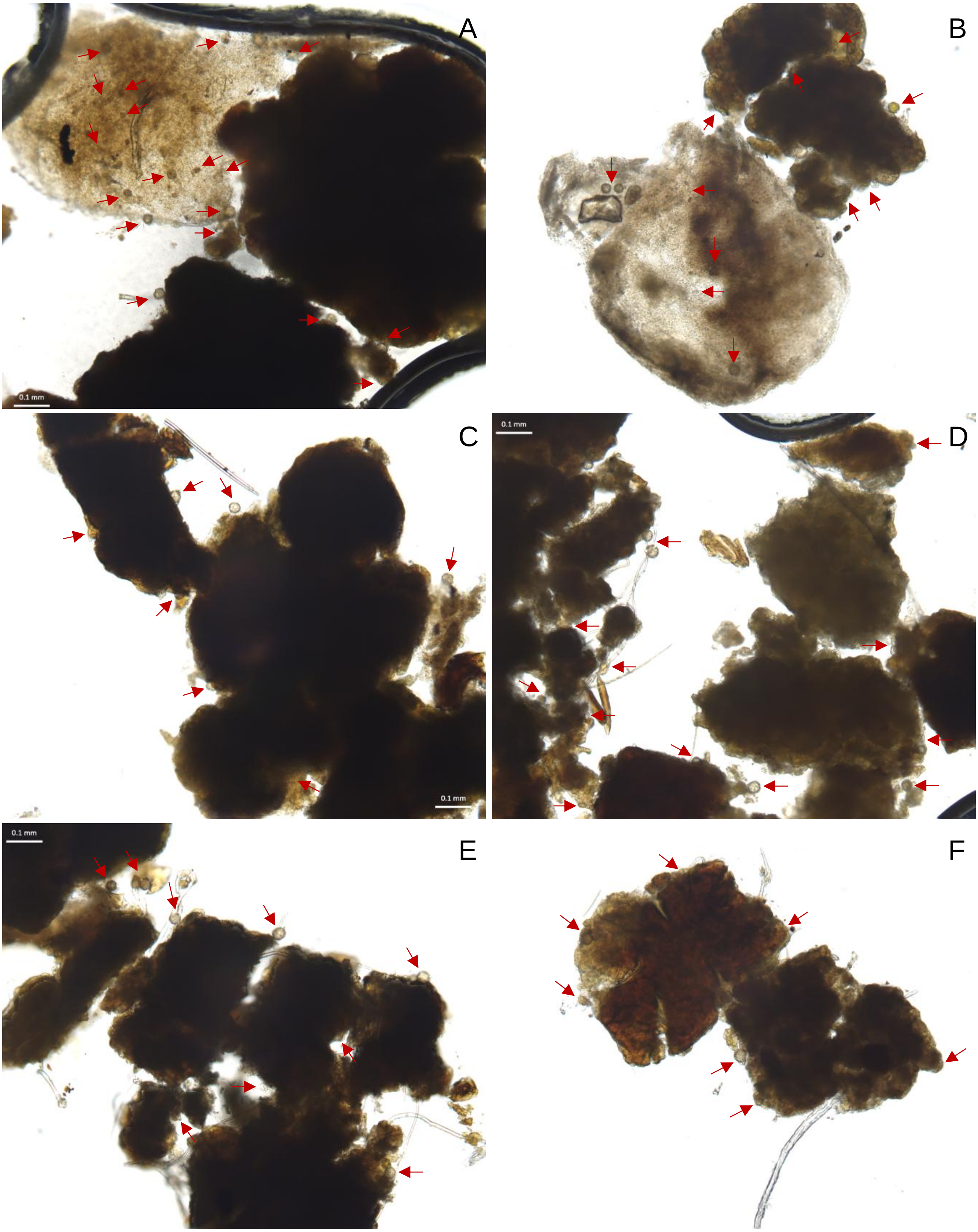

Supplementary fig. 2. Droppings from six captured *Isoplia lasiosoma* beetles reveal clearly visible pollen grains (red arrows) of plant species co-flowering with *Disa similis* in Red Desert Nature Reserve and Rennie's Beach (KZN, SA) under a compound microscope.
Scale: 0.1 mm.
